# Smartphone Pedometer Sensor Application for Evaluating Disease Activity and Predicting Comorbidities in Patients with Rheumatoid Arthritis: A Validation Study

**DOI:** 10.3390/s22239396

**Published:** 2022-12-02

**Authors:** Stefan R. Wagner, Rasmus R. Gregersen, Line Henriksen, Ellen-Margrethe Hauge, Kresten K. Keller

**Affiliations:** 1 Department of Electrical and Computer Engineering, Aarhus University, 8200 Aarhus, Denmark; 2 Department of Rheumatology, Aarhus University Hospital, 8200 Aarhus, Denmark; 3 Department of Clinical Medicine, Aarhus University, 8000 Aarhus, Denmark

**Keywords:** rheumatoid arthritis, pedometer, activity tracker, smartphone, comorbidity

## Abstract

Smartphone-based pedometer sensor telemedicine applications could be useful for measuring disease activity and predicting the risk of developing comorbidities, such as pulmonary or cardiovascular disease, in patients with rheumatoid arthritis (RA), but the sensors have not been validated in this patient population. The aim of this study was to validate step counting with an activity-tracking application running the inbuilt Android smartphone pedometer virtual sensor in patients with RA. Two Android-based smartphones were tested in a treadmill test-bed setup at six walking speeds and compared to manual step counting as the gold standard. Guided by a facilitator, the participants walked 100 steps at each test speed, from 2.5 km/h to 5 km/h, wearing both devices simultaneously in a stomach pouch. A computer automatically recorded both the manually observed and the sensor step count. The overall difference in device step counts versus the observed was 5.9% mean absolute percentage error. Highest mean error was at the 2.5 km/h speed tests, where the mean error of the two devices was 18.5%. Both speed and cadence were negatively correlated to the absolute percentage error, which indicates that the greater the speed and cadence, the lower the resulting step counting error rate. There was no correlation between clinical parameters and absolute percentage error. In conclusion, the activity-tracking application using the inbuilt Android smartphone pedometer virtual sensor is valid for step counting in patients with RA. However, walking at very low speed and cadence may represent a challenge.

## 1. Introduction

Disease activity and impact of disease in rheumatoid arthritis (RA) are typically measured with questionnaires such as the health assessment questionnaire disability index (HAQ-DI) or composite scores such as the disease activity score (DAS28) [1,2]. These methods are typically performed only at hospital visits and therefore risk missing signs of increased disease activity that occurs between visits. Also, self-reported information about physical activity can be biased due to the risk of reporting error [3].

Measuring physical activity continually may assist in the early discovery of relapses [4]. In addition, cardiovascular and pulmonary mortality are elevated in patients with RA compared to the general population [5,6]. Measuring physical activity may be relevant for predicting the risk of developing comorbidities such as cardiovascular disease [7].

Dedicated activity tracker devices developed for research can measure physical activity in healthy individuals [8]. Recently this type of device was validated in RA patients to investigate physical activity [9]. A range of potential issues related to long-term tracking of patients exists with such advanced activity trackers. Firstly, they can only be carried for a limited period of time before they need recharging or offloading of data. Next, they are expensive to obtain and maintain, which makes them unsuitable for large long-term cohort studies. A low-cost alternative to this is to use the physical activity data from the participants’ personal smartphones using a dedicated and secure activity tracker application (app) utilizing the inbuilt smartphone pedometer virtual sensor. This incurs no added costs for equipment, assuming participants already have a phone with an internet connection, and can arguably be feasible to use for long-term clinical trials. This method may be reliable in healthy individuals [10], but the validity has never been tested in patients with RA and other diseases and conditions that impact mobility. This could be important since the walking pattern in patients with RA could likely be different from that of healthy individuals [11].

The aim of this study was, for the first time, to validate step counting with an activity tracker app running the inbuilt Android smartphone pedometer in patients with RA.

## 2. Materials and Methods

### 2.1. Study Population and Recruitment

Patients with RA were recruited at a planned visit in the outpatient clinic at the Department of Rheumatology, Aarhus University Hospital, Denmark. Inclusion criteria were: (1) diagnosis of RA, (2) at least 18 years of age, (3) able to provide written informed consent. Clinical data relevant for characterization of patients with RA, including sex, age, disease duration, prosthetic joint, previous fractures, erosive disease, anti-citrullinated peptide antibody (ACPA) status, IgM rheumatoid factor (RF) status, and clinical data relevant for characterization of disease activity in patients with RA and thereby the ability to walk, including swollen joints, tender joints, HAQ-DI, DAS28-CRP, and visual analogue score (VAS) pain, were obtained by KKK from the Electronic Patient Journal of Central Denmark Region and the nationwide quality registry for patients with rheumatic diseases [1,2,12]. All subjects gave written informed consent. The study was assessed by the Central Denmark Region Committees on Health Research Ethics and did not need ethical approval. Data were collected anonymously.

### 2.2. Experimental Design

The study was conducted using a treadmill (Kilberry PMT-4550, Shanghai, China) in a laboratory setting at the Department of Rheumatology, Aarhus University Hospital. During the test, patients carried two Android smartphones in a waist pouch at the front right hip to ensure equal placement. A Google Pixel 4 Android smartphone (Google Inc., Mountain View, CA, USA) and a Samsung Galaxy A02 Android smartphone (Samsung, Seoul, Republic of Korea), both running Android 10, were used.

The two study smartphones had the custom-made pedometer application BeSafe installed, which was developed by the authors (Department of Electrical and Computer Engineering, Aarhus University, Denmark), receiving step information from the Android Step counter virtual sensor application programming interface (API), which was introduced as part of Android SDK 19, provided by Google (Google Inc., Mountain View, CA USA).

Data was sent in soft real time to a computer using a WiFi network data collection platform. BeSafe was built to allow for full control of the data collection and distribution process. BeSafe was made using the Xamarin C# Android framework and developed using Visual Studio 2019, both provided by Microsoft (Microsoft Corporation, Redmond, WA, USA), and was built using the Android 29 SDK targeting Android version 10 and higher (Google Inc., Mountain View, CA, USA).

In theory, BeSafe can be replaced by any third-party app running the same Android step counter virtual sensor, as long as the data can be collected during or after the experiment. Thus, this should arguably support the generalizability of the results.

The BeSafe pedometer application collected step data automatically throughout the entire test using a WiFi connection. Parallel with the automatic registration manual, step counting was performed in real time by the observers and registered on a per-step basis on the testbed computer by clicking a keyboard button. Patients walked 100 steps at 6 different speeds from 2.5 km/h to 5 km/h.

The treadmill’s digital speed settings ascertained that constant speed was maintained by the participants. For each speed, and when starting a new experiment, there would be a short transition phase until a steady state was reached. Thus, the observers manually registered the steps by clicking a button on the automated data collection unit, deferring manually observed step registrations until steady state was reached for each speed. The data collection unit would, in real time, receive the counted steps from the two smartphones and automatically couple this with the manually observed step counts. Please find images of the testbed setup in the supplementary materials section as Supplementary Figure S1.

A typical way of gaining access to activity data generated by a smartphone is to use the cloud services of the vendor, such as: Google, US, Apple, US, Fitbit, US, Garmin, US, Samsung, South Korea, and other vendors. In this type of scenario, the user is asked to allow for activity data to automatically be uploaded to a cloud service, from where the user themselves can later download the data. This data-handling method also allows third parties to retrieve the data, typically using a secure Web API under the control of the vendor, and usually works well for personal usage, given informed consent of the user to the cloud vendor. However, this method is not always acceptable for clinical trials, as data that is hosted by an unknown third party at an unknown hosting location is often considered insecure and with the risk of being forwarded to other entities beyond the investigator’s control. The problem being that trial data is then kept by a third party beyond the control of the investigator’s organization. Although current data protection regulations, for example, the General Data Protection Regulation (GDPR) in Europe, do allow for such scenarios, they are often not considered relevant to use in clinical trials due to local safety regulations. Instead, in our approach, we use the same on-phone sensors as used by Google and other app vendors, but we omit using the Google cloud services, and instead use our own secure data distribution and data-hosting infrastructure, where data is stored at a secure university server. Thus, safety is achieved by using a combination of hashed identification values for the participants and secure web tunneling using transport layer security (TLS) protocol and Hypertext Transfer Protocol Secure (HTTPS), thus keeping a strict secure encrypted communication tunnel for the data. In other words, data are neither stored on the smartphone itself, nor on any intermediary server. Data are sent directly to a clinical trial team-controlled server, which is again placed within a secure server facility at the university server park.

### 2.3. Statistics

Data was analyzed using STATA (version 17.0, StataCorp., College Station, TX, USA) and JASP Statistical Software (University of Amsterdam, Amsterdam, The Netherlands). Normality was measured using histogram, QQ plot, and the Shapiro–Wilks test. If data was not normally distributed, non-parametric tests were performed. A *p*-value less than 0.05 was considered statistically significant.

Absolute percentage error (APE) values were calculated as the number of manually counted steps minus the number of steps counted by the Samsung or Pixel phone and divided by the number of manually counted steps. APE and mean absolute percentage error (MAPE) for all patients were calculated for all speed and cadence categories. An overall APE and MAPE of qualified data from all speeds was also calculated. Cadence was determined for every complete iteration as the number of observed steps divided by the completion time.

The paired APE values of the Samsung phone and the Pixel phone were compared with the non-parametric Wilcoxon signed-rank test. Spearman’s rank correlation of APE, walking speed, cadence, and observed steps were calculated. Continuous clinical data were correlated with overall APE and APE at each speed using Spearman’s rank correlation. Comparisons between dichotomous clinical data and overall APE and APE at each speed were made using the Mann–Whitney test.

## 3. Results

A total of 30 patients with established RA were included, and parameters relevant to the gait were evaluated, as indicated in Table 1. Patients had a long disease duration, with a median of 13 years, and low disease activity, with a median DAS-28 of 2.2.

**Figure 1:**
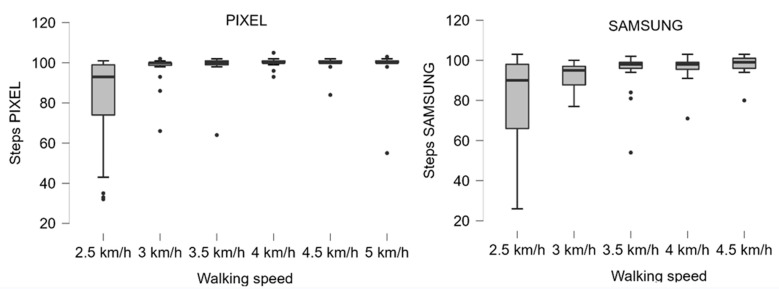
Box plot of the number of steps for both devices at the six different speeds. Median and interquartile range are demonstrated.

Of the 30 patients recruited, one participant did not manage to complete the treadmill walking test at 2.5 km/h, whereas 29 patients participated in up to 6 tests per patient in the range of 2.5 to 5 km/h, depending on their ability to walk. A total of 162 tests were performed, including 2 tests discarded due to failed recording, resulting in a total of 16,009 recorded observed steps. In total, the Samsung phone recorded 14,961 steps and thus incurred an undercount of 6.5%, while the Pixel phone recorded 15,364 steps with an undercount of 4.0%. The distribution of steps at various walking speeds is demonstrated in Figure 1, showing that the lowest speed of 2.5 km/h is associated with a high undercount for both phones.

MAPE of the number of steps for both phones was 5.9%, ranging from 7% for the Samsung phone to 4.8% for the Pixel phone. MAPE for the different walking speeds for the Samsung phone ranged from 19.3% at 2.5 km/h to 1.5% at 5 km/h (Table 2). MAPE for the Pixel phone ranged from 17.7% at 2.5 km/h to 1% at 4 km/h (Table 2). Overall, APE was larger for the Samsung phone compared to the Pixel phone (*p* < 0.01).

Correlations between observed steps, device steps, cadence, walking speed, and device APE are demonstrated in Table 3. A positive correlation between Samsung APE and Pixel APE across all walking speeds was found (r = 0.3, *p* < 0.001). Likewise, there was a positive correlation between step and speed for both Samsung (r = 0.5, *p* < 0.01) and Pixel phones (r = 0.5, *p* < 0.01). Furthermore, there was a positive correlation between step and cadence for the Samsung (r = 0.4, *p* < 0.01) and Pixel phones (r = 0.3, *p* < 0.01). A negative correlation was seen between speed and device APE for the Samsung phone (r = −0.4, *p* < 0.001) and the Pixel phone (r = −0.4, *p* < 0.001). Finally, there was a negative correlation between cadence and device APE for the Samsung phone (r = −0.4, *p* < 0.001) and the Pixel phone (r = −0.3, *p* < 0.001).

There was no correlation between disease duration, HAQ-DI, VAS-pain, DAS28-CRP, and the overall APE or APE at 2.5 km/h (Table 4). There was no correlation for the other speeds (data not shown).

Likewise, there was no statistical difference between overall APE or APE at 2.5 km/h concerning ACPA status, RF status, and erosive status (Table 5). There was no statistical difference for the other speeds (data not shown).

## 4. Discussion

The validity of wearable activity trackers has previously been validated in several studies, which all found a good agreement at most frequently used walking speeds, while very low walking speeds remain a challenge for most devices, as also found in our study [13,14,15,16,17,18,19]. However, to the best of the authors’ knowledge, no studies have examined the use of smartphone-based pedometers for telemedicine use in RA patients, although smartphone-based applications have the potential to allow for valid, low-cost, long-term monitoring. Several studies have found that smartphone-based systems are useful for telemedicine applications for other chronic patient groups, including hypertension [20] and cancer [21].

Activity-tracking devices have recently been introduced to measure physical activity in randomised trials in patients with RA [22]. A study has also demonstrated an association between patient-reported outcome measures such as HAQ-DI and steps measured with an activity tracker device, indicating that measured steps may reflect the wellbeing of the patient, but the device was not validated for use in patients with RA [23]. Likewise, a study demonstrated that machine-learning models of steps counted with an activity tracker device could predict self-reported disease activity in patients with RA [4,24]. Activity trackers may therefore have large perspectives for monitoring disease activity in patients with RA. However, only a few studies have actually investigated the validity of activity-tracking devices in patients with RA. A recent study using the activPAL activity- tracking device in patients with RA concluded that it was not valid for measuring steps due to a significant step underestimation of 26% [25]. Another study that used three different activity tracker devices found inferior validity in patients with RA compared to a population of younger healthy controls [26]. A third study demonstrated good validity for evaluating sedentary, standing, and walking time in patients with RA [9]. In this study, the smartphone activity-tracking app proved to be valid for measuring steps in patients with established RA, since only a very small variation compared to the gold standard of manual counting was found [27]. An advantage of the smartphone-based activity-tracking app used in this study compared to traditional activity tracker devices is that they can be used for a long period of time compared to a decrease in adherence over time seen with traditional activity-tracking devices [28]. This may be necessary for measuring disease activity continually, and possibly also as a predictor of future cardiovascular morbidity [7]. Interestingly, an earlier study demonstrated that RA patients underestimate their sedentary time, underscoring the importance of objective measures of activity [3].

Most variation was seen at the lowest speed, where the devices may not provide valid results. However, this represents an unnaturally slow speed for most, and it could be argued that not many patients would be expected to walk at this speed. Still, the speed is relevant, as slow speed could likely occur in a domestic setting, doing house chores, and other activities of daily living (ADL) [29]. At higher speeds the small variation could likely be attributed to experimental bias, e.g., when the first and last step are counted. However, both walking speed and cadence correlated negatively to APE and were therefore important for pedometer validity. This is in accordance with previous studies in healthy individuals using smartphone-based as well as dedicated pedometers [10,13,30]. The overall MAPE of both the Samsung and Pixel devices were small, and thus both devices are suitable for use. The Pixel represents a high-level and high-cost device, while the Samsung represents a low-cost device. Still, the difference between them does not seem clinically relevant.

Previous studies have demonstrated that positioning of the device may be of importance for accuracy. Waist-worn pedometers, as used in this study, may be more reliable compared to arm and hand-worn pedometers for walking [14]. Song et al. found that different carrying positions require different algorithms in order to provide optimal accuracy, which they found was not achieved with commercial pedometer applications at self-paced walking speeds under different smart phone carrying positions [31].

There were limitations in this study. First, the majority of patients were in clinical remission, and results could therefore be different in active disease. Secondly, a control group with healthy individuals was not used. Thirdly, only two Android smartphones from two different vendors were tested, and results may therefore not be applicable to other smartphones. We will elaborate further on this in the following section. Fourthly, this study did not investigate whether RA patients actually do carry their phones for a sufficient timespan during the day to provide a valid sample for assessing the daily activity levels, nor how they carry their phones, which could be an issue, according to Song et al. [31].

In addition, real-world activities in different settings and on different types of floors and surfaces are potentially different from treadmill-based step patterns, including doing office work, travelling by bus, riding a bicycle, driving a car, vacuum cleaning, cooking, toileting, and washing. Ebara et al. assessed different models of smartphones with the same Android operating system during office work, while travelling on a bus, and driving a car [32]. The Ebara et al. study found that the false detection rates of step-counting sensors were low when subjects were sitting during office work, but false detection would increase depending on the smartphone model while riding the subway or driving a car. The authors conclude that it is possible that physical activity may be overestimated, and physical inactivity may be underestimated when attempting to make assessments with smartphone step-counting sensors only.

These findings lead us to consider that smartphone-based activity trackers should contain automated calibration features, allowing for an initial 100-step walking test at the patient’s own pace and with the preferred placement of the patient during physical consultations for calibration and validation purposes, as well as a series of relevant everyday activities for simulating real-world activities. Also, we propose to investigate automated error detection features, as also suggested by Ebara et al. [32].

The choice of validation smartphone devices: the Samsung Galaxy Model A02, Samsung Cororation, Seoul,.South Korea, and the Pixel model 4, Google Inc., Mountainview, CA, USA, both running Android version 10, is a further limitation of the study. While Samsung, holds approximately 22% of the market, according to 2022 market statistics from Counterpoint research, US, it is not assumed that the exact same accelerometer (or with the same characteristics) will be used in future models, or even models produced the same year.

In addition to this, other models from vendors such as Apple Inc., Cupertino, CA, USA (market share of 11%) and Xiaomi Inc., Beijing, China (market share of 13%) would also be highly relevant to validate as part of future work, but the same issue persists, that the sensor itself may change even within the same series. As there are many different configurations of makes and models, and even potential differences in operating system versions, it could also be relevant to perform a personalized validation of all new participants’ devices as they are onboarded in a large-scale clinical trial. Thus, it could be relevant to initially ask a new user being onboarded, to walk 100 steps at the user’s own pace in order to perform a personalized baseline validation. Again, a full treadmill test for each future user would be relevant, but this is not feasible with the full trial protocol used in the current study, as the level of preparation and length of the testing would not be feasible for real-world clinical use.

The BeSafe app was designed for this study to allow for independent and secure data collection and data storage, ensuring that activity data are only kept at the hosting organizations dedicated servers and are only available for the involved clinicians and the patients themselves. Thus, even though previous studies have found that a majority of users would be willing to share activity data for research use [33], it could be argued that user data should not be shared with any commercial or third-party company, including Google, Apple, Fitbit, Garmin, and other commercial vendors unless full user consent is provided.

## 5. Conclusions

In conclusion, this is the first study demonstrating that an activity tracking-app running the inbuilt Android smartphone pedometer virtual sensor was valid in patients with RA, except for very low walking speed and cadence.

The next step will be field tests evaluating the setup, including active measured time in patients with RA on their own smartphone models. We need to evaluate the performance of the app under real-world conditions, at home, during commute, and at work, to understand how the results of this study, which were under ideal laboratory conditions, compare with real-world situations. We therefore expect a much lower validity in real-life situations but would like to understand whether results would still be usable as a clinical parameter and indicator of patient disease progression.

Finally, we recommend that automated error detection and calibration in step counting is investigated further.

## Figures and Tables

**Table 1:** Demographics and laboratory and clinical characteristics of patients with rheumatoid arthritis.

Parameter	RA Patients (n = 30)
Female, n/total (%)	22/30 (73)
Years of age, median (IQR)	61 (50–74)
Years since diagnosis, median (IQR)	13 (4–21)
Prosthetic joint in LE, n/total (%)	4/30 (13)
Previous fractures in LE since diagnosis, n/total (%)	1/30 (3)
Erosive disease, n/total (%)	21/30 (70)
Anti-citrullinated peptide antibody positive, n/total (%)	21/28 (75)
IgM Rheumatoid factor positive, n/total (%)	21/30 (76)
Swollen joints UE, n/total (%)	6/30 (20)
Tender joints UE, n/total (%)	8/30 (27)
Swollen joints LE, n/total (%)	1/30 (3)
Tender joints LE, n/total (%)	5/30 (17)
DAS28-CRP, median (IQR)	2.2 (1.6–2.9)
HAQ-DI, median (IQR)	0.2 (0–0.9)
VAS pain (0–100), median (IQR)	21 (7–59)

IQR = interquartile range; UE = upper extremities; LE = lower extremities; DAS = disease activity score; HAQ-DI = Health Assessment Questionnaire-Disability Index; VAS = visual analogue scale.

**Table 2:** The average percentage error of the Samsung and Pixel devices split into the 6 test speeds.

	APE Samsung (km/h)						APE Pixel (km/h)					
	2.5	3	3.5	4	4.5	5	2.5	3	3.5	4	4.5	5
Valid	29	28	26	27	25	25	29	28	26	27	25	25
Mean	19.3	7.3	5.3	4.1	2.9	1.5	17.7	2.6	2.4	1.0	1.1	2.5
Median	10.0	5.0	2.0	2.0	2.0	1.0	7.0	1.0	1.0	1.0	0.0	1.0
Minimum	0.0	0.0	0.0	0.0	0.0	0.0	0.0	0.0	0.0	0.0	0.0	0.0
Maximum	74.0	23.0	46.0	29.0	20.0	5.0	68.0	34.0	39.0	7.0	16.0	45.0

APE = average percentage error.

**Table 3:** Spearman’s rank correlation analysis results showcasing correlations of the study variables.

Variable		Observed Steps	Steps Samsung	Steps Pixel	Cadence	Walking Speed km/h	APE Samsung	APE Pixel
1. Observed Steps	Spearman’s rho	—						
	*p*-value	—						
2. Steps Samsung	Spearman’s rho	0.033	—					
	*p*-value	0.680	—					
3. Steps Pixel	Spearman’s rho	0.141	0.313	—				
	*p*-value	0.075	<0.001	—				
4. Cadence	Spearman’s rho	0.113	0.392	0.326	—			
	*p*-value	0.156	<0.001	<0.001	—			
5. Walking speed km/h	Spearman’s rho	0.162	0.450	0.459	0.511	—		
	*p*-value	0.040	<0.001	<0.001	<0.001	—		
6. APE Samsung	Spearman’s rho	−0.021	−0.889	−0.287	−0.385	−0.459	—	
	*p*-value	0.795	<0.001	<0.001	<0.001	<0.001	—	
7. APE Pixel	Spearman’s rho	0.004	−0.253	−0.318	−0.249	−0.343	0.349	—
	*p*-value	0.962	0.001	<0.001	0.001	<0.001	<0.001	—

APE = absolute percentage error of steps.

**Table 4:** Spearman’s rank correlation between APE and continuous clinical variables.

	**Overall APE**			
	**Samsung Device**		**Pixel Device**	
	**R**	***p***	**R**	***p***
Years since diagnosis	−0.28	0.14	−0.09	0.64
HAQ-DI	−0.22	0.33	0.03	0.90
VAS pain (0–100)	−0.02	0.93	0.01	0.97
DAS-28 CRP	−0.19	0.40	−0.07	0.76
	**APE 2.5 km/h**			
	**Samsung Device**		**Pixel Device**	
	**R**	***p***	**R**	***p***
Years since diagnosis	−0.26	0.17	−0.13	0.47
HAQ-DI	−0.29	0.19	−0.13	0.57
VAS pain (0–100)	−0.08	0.71	−0.00	0.99
DAS-28 CRP	−0.25	0.27	−0.05	0.83

DAS = disease activity score; HAQ-DI = Health Assessment Questionnaire-Disability Index; VAS = visual analogue scale.

**Table 5:** Comparison between APE and dichotomous clinical variables.

	**ACPA Positive** **(n = 20)**	**ACPA Negative** **(n = 7)**	***p***
Overall APE for Samsung device	5.7 (2.6–11)	3.7 (2.3–10.2	0.65
Overall APE for Pixel device	5.0 (1.1–9.2)	0.7 (0.3–2.2)	0.05
APE 2.5 km/h for Samsung device	19 (2.5–38)	7.0 (3.0–26)	0.35
APE 2.5 km/h for Pixel device	11 (2.0–41)	1.0 (1.0–7.0)	0.05
	**RF Positive** **(n = 20)**	**RF Negative** **(n = 9)**	***p***
Overall APE for Samsung device	5.1 (2.4–9.7)	7.7 (3.0–11.7)	0.32
Overall APE for Pixel device	1.8 (0.9–7.4)	2.2 (0.7–11.8)	0.97
APE 2.5 km/h for Samsung device	9.5 (2.0–31.5)	13 (3.0–44)	0.40
APE 2.5 km/h for Pixel device	16.8 (0.5–33)	7.0 (1.0–24)	0.72
	**Erosive** **(n = 21)**	**Non-Erosive** **(n = 8)**	***p***
Overall APE for Samsung device	4.8 (2.3–10.2)	5.7 (4.1–10.6)	0.58
Overall APE for Pixel device	1.2 (0.8–6.5)	7.3 (2.0–10.9)	0.11
APE 2.5 km/h for Samsung device	9.0 (2.0–34)	11.5 (2.5–30.5)	0.76
APE 2.5 km/h for Pixel device	3.0 (1.0–26)	8.5 (5.0–35)	0.39

## Data Availability

The datasets used during the current study are available from the corresponding author on reasonable request.

## Supplementary Materials

The following supporting information can be downloaded at: https://www.mdpi.com/article/10.3390/s22239396/s1, Figure S1: Collage of the experimental setup.
