# Supplementary material for: Smartphone Pedometer Sensor Application for Evaluating Disease Activity and Predicting Comorbidities in Patients with Rheumatoid Arthritis: A Validation Study
Source: Sensors (Basel). 2022 Dec 2;22(23):9396. doi: 10.3390/s22239396 (PMC9735816; doi:10.3390/s22239396)
Supplement: Supplementary file 1 [file sensors-22-09396-s001.zip › sensors-2020325-supplementary.pdf]

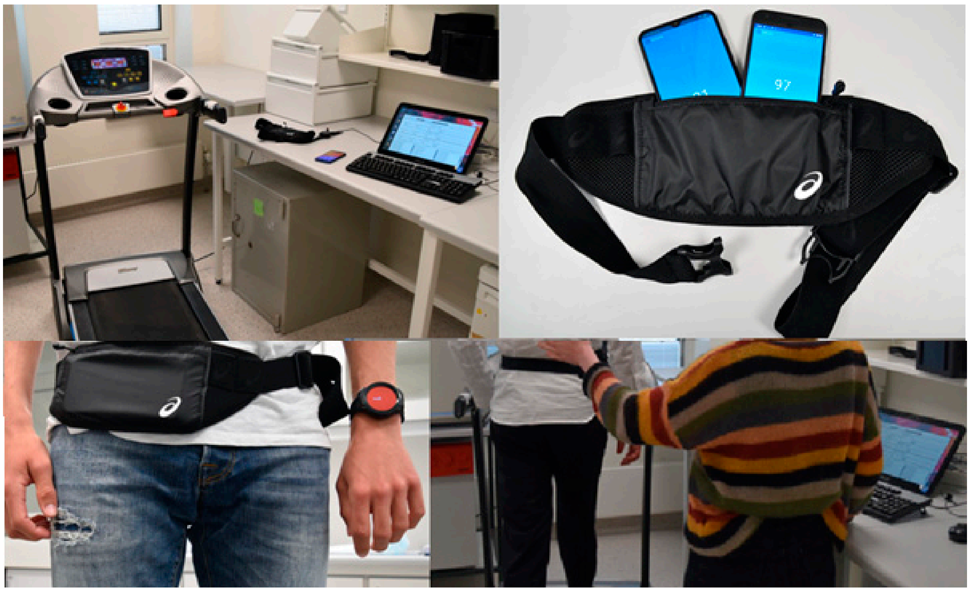

Supplementary Figure S1: Top left, the treadmill which allows for data collection at fixed walking speeds. Top middle, the data collection software, which automatically receives the step count from both phones and allows the observer to manually register observed steps. When reaching 100 steps at a certain speed, the data registration stops automatically, and calculates the differences needed for statistical analysis. Top right the pocket pouch with the two used smartphone models running BeSafe. Bottom left, the pouch affixed to the stomach of the test participants ensuring a standardized measurement point. Bottom right: the test facilitator ensures that the participant is walking steadily and unsupported, ready to stop the experiment if the speed proves too strainful for the participant. Also, the facilitator decides when a steady speed is established, and only when the facilitator starts registering observed steps, by pressing the keyboard will the observed and two measured steps be recorded in real time.
